# A Quantum-Based Similarity Method in Virtual Screening

**DOI:** 10.3390/molecules201018107

**Published:** 2015-10-02

**Authors:** Mohammed Mumtaz Al-Dabbagh, Naomie Salim, Mubarak Himmat, Ali Ahmed, Faisal Saeed

**Affiliations:** 1 Faculty of Computing, Universiti Teknologi Malaysia, Skudia 81310, Malaysia; E-Mails: naomie@utm.my (N.S.); barakamub@yahoo.com (M.H.); alikarary@gmail.com (A.A.); faisalsaeed@utm.my (F.S.); 2 Faculty of Engineering, Karary University, Khartoum 12304, Sudan

**Keywords:** quantum mechanics, quantum-based similarity, complex numbers, similarity searching approach, ligand-based, virtual screening

## Abstract

One of the most widely-used techniques for ligand-based virtual screening is similarity searching. This study adopted the concepts of quantum mechanics to present as state-of-the-art similarity method of molecules inspired from quantum theory. The representation of molecular compounds in mathematical quantum space plays a vital role in the development of quantum-based similarity approach. One of the key concepts of quantum theory is the use of complex numbers. Hence, this study proposed three various techniques to embed and to re-represent the molecular compounds to correspond with complex numbers format. The quantum-based similarity method that developed in this study depending on complex *pure* Hilbert space of molecules called Standard Quantum-Based (SQB). The recall of retrieved active molecules were at top 1% and top 5%, and significant test is used to evaluate our proposed methods. The MDL drug data report (MDDR), maximum unbiased validation (MUV) and Directory of Useful Decoys (DUD) data sets were used for experiments and were represented by 2D fingerprints. Simulated virtual screening experiment show that the effectiveness of SQB method was significantly increased due to the role of representational power of molecular compounds in complex numbers forms compared to Tanimoto benchmark similarity measure.

## 1. Introduction

Virtual screening refers to the use of a computer-based method to process compounds from a library or database of compounds in order to identify and select the ones that are likely to possess a desired biological activity, such as the ability to inhibit the action of a particular therapeutic target. The selection of molecules with a virtual screening algorithm should yield a higher proportion of active compounds, as assessed by experiment, relative to a random selection of the same number of molecules [1,2].

Many virtual screening (VS) approaches have been implemented for searching chemical databases, such as substructure search, similarity, docking and QSAR [3]. Of these, similarity searching is the simplest, and one of the most widely-used techniques for ligand-based virtual screening (LBVS) [4]. The increasing of the importance of similarity searching applications is particularly due to its role in lead optimization in drug discovery programs, where the nearest neighbors for an initial lead compound are sought in order to find better compounds.

There are many studies in the literature associated with the measurement of molecular similarity [5,6,7]. Similarity searching aims to search and scan chemical databases to identify those molecules that are most similar to a user-defined reference structure using some quantitative measures of intermolecular structural similarity. However, the most common approaches are based on 2D fingerprints, with the similarity between a reference structure and a database structure computed using association coefficients such as the Tanimoto coefficient [2,8]. Several methods have been used to further optimize the measures of similarity between molecules, including weighting, standardization, and data fusion [9,10,11,12,13].

Similarity measures methods play a significant role in detecting the rate for pairwise molecular similarity. In this study, the similarity method that developed inspired from quantum machines theory. The quantum machines was recently employed in the information retrieval field [14,15,16], and due to many similarities aspects between the text and chemical information retrieval, it was adapted for chemoinformatics as well [17]. These analogies have provided the basis for the work in this paper, which is to introduce a new similarity method inspired from quantum theory to calculate the similarity for chemical database according to the reference structures. The Standard Quantum-Based (SQB) similarity method requires Re-representation of molecular compounds in order to be adapted with the mathematical quantum space which is called complex Hilbert space. The representation of molecular compounds formulates in complex numbers formats which can play vital role in development of SQB method. The use of complex numbers is one of the key concepts of the mathematical formalism of quantum theory. This study also developed three different techniques to re-represent and embed the molecular compounds in complex numbers formats. Finally, the screening experiment was simulated with three popular datasets converted to Pipeline Pilot ECFC_4 2D fingerprints, which are MDL Drug Data Report (MDDR), Maximum Unbiased Validation (MUV) and Directory of Useful Decoys (DUD).

## 2. Related Work

In a broad sense, the comprehensive umbrella of chemoinformatics is not restricted to molecular searching and drug design, but includes several classical chemical disciplines such as physical chemistry, medicinal chemistry, analytical chemistry, and others. Hence, the interaction among disciplines plays a fundamental role in generating new approaches to deal with chemoinformatic issues such as quantum-chemistry approaches to deal with mathematical aspects of chemistry. Generally during chemical bonding, there is no substitute to identify the behavior of atoms than quantum-chemistry approaches. This has led several scientists to consider only molecular descriptors derived from quantum chemistry. The quantum-chemical descriptors can be classified depending on the type of descriptors into orbital-based, energy-based, wave function based, and others [18].

The bridge between chemical concepts and quantum-chemistry was introduced by Atoms-in-Molecules (AIM) quantum approach [19]. The quantum-chemical algorithms in QSAR/QSPR play a fundamental role in providing higher accuracy than Force-Field based methods. Holder, *et al.* [20] employed quantum chemical concepts first for verification of graph theory of molecules, and second to predict the refractive index of polymer matrices. The quantum-chemistry descriptors in the development of QSAR/QSPR deal with the chemical, physical, biochemical, and pharmacological properties of compounds [21,22]. The combination of two types of connectivity 2D and 3D of molecular descriptors with quantum-chemistry descriptors showed a more preferable approach to QSPR than using each connectivity individually [23]. Within the same environment, the approaches of quantum-chemical are also used with 3D-QSAR models to calculate stereo-electronic properties [24,25,26]. Another study [27] employed quantum-chemistry depending on some properties of orbital calculations of molecules in order to overcome the limitation of classical QSAR approaches.

On the other hand, the nature of quantum mechanics as well as computing power were also derived and used in docking and improvement of known lead compounds in order to provide highest accuracy [28,29,30]. In addition, Quantum Mechanics Methods (QMM) were extensively applied in Linear Scaling in order to evaluate the binding enthalpy between ligand and proteins [31,32,33,34]. Other usage of QMM was to calculate energies and optimization of molecular structures at semi-empirical level [35]. In drug discovery process, the QMM has been devoted to investigate and describe electronic properties of molecules such as electronic and polarize effects, charge distribution, and bond state (forming/breaking) [36,37].

One of the approaches that was used for compounds similarity searching is Molecular Quantum Similarity Measure (MQSM) which was proposed by Carbó [38]. The MQSM approach depends on quantum-chemical descriptors to measure the similarity/diversity of molecules through the analysis of electron density function which is calculated by QMM. As a consequence, the QSAR models were developed based on MQSM [39,40,41]. In addition, the Molecular Quantum Self-Similarity Measures (MQSSM) was developed based on MQSM by comparing each molecule with itself [37,42,43]. The MQSM was also employed to classify the quantum objects of molecules by using dendrograms [44].

Moreover, Maldonado *et al.* [7] introduced comprehensive study of the applications and theories of molecular similarity measures in chemoinformatics. Generally, the measures of molecular similarity rely mainly on three factors. First are the features of molecular structures which can be used to detect the similarity/diversity of molecular compounds, which is known as descriptor. The molecular descriptors differ from 1D, 2D, and 3D molecular structures. The molecular descriptors influence the chemical, physical and biological properties as well as the order of atoms and chemical bonds [5,7]. Second, the similarity coefficients, which are mostly categorized to distance, correlation and probabilistic coefficient, for instance, Tanimoto, Euclidean distance, Pearson and so on. More details about similarity coefficients are discussed in the recent comprehensive study presented by Todeschini *et al.* [8]. The third and last is the degree of importance for molecular fragments represented by weighting schema approaches. Various techniques were used for this purpose such as Bayesian Inference Network [11].

Evident from the above studies, the Quantum Mechanics Methods were used at semi-empirical level of computational chemistry whether for quantum-chemical descriptors or quantum-similarity measure. In this study, the adoption of quantum mechanics concepts is investigated to be used as similarity searching method to find similarity/dissimilarity between reference and library molecules.

## 3. Methods

### 3.1. Quantum Model

Each similarity measure is made up of two elements: a mathematical representation of the relevant molecular information (*i.e.*, vectors, graphs, functions) and some form of index or coefficient compatible with the representation. Similarity coefficients can be classified into correlation, associative, distance coefficients, and probabilistic [7,10,45]. The quantum-based similarity methods inspired from quantum probability formalism which can be considered a geometric generalization of standard probability theory that makes use of Hilbert space, subspace and unit vectors. On the other hand, quantum physics offers probabilistic, logic and geometric formalism based on mathematics of Hilbert space to describe the behavior of matter at atomic and subatomic scales. In this paper, we employed the concepts of quantum mechanics theory at two levels. Firstly, creating quantum framework in virtual screening by representation of molecular compounds and references based on complex Hilbert space via three proposed embedded techniques as well as real Hilbert space as special case of representation. Second, presenting a new similarity method, namely Standard Quantum-Based (SQB), based on quantum model components. The following subsections presented more details about the quantum model components.

#### 3.1.1. Creating Molecular Compounds Subspace

The mathematical representation of molecular information is a non-trivial task, and it plays a fundamental and significant role for similarity measure. Therefore, the molecular compounds should be reformulated for adapting to quantum mechanics. The probabilistic formalism of quantum relies on a multidimensional representation of objects to provide more powerful way to tackle the challenges. The probabilistic events are represented as subspace in a Hilbert space. The latter can be an extended version (*i.e.*, infinite-dimensional space) of the notion of Euclidean space. The subspace is spanned by the basis vectors. All these components can be finished with geometry of chemical-information space. Moreover, the inner product gives the geometry which can be used to derive the probability of similarity between reference structure and library compounds. Therefore, the strong connection between probabilities and geometry presents in the quantum probability formalism.

**Figure 1:**
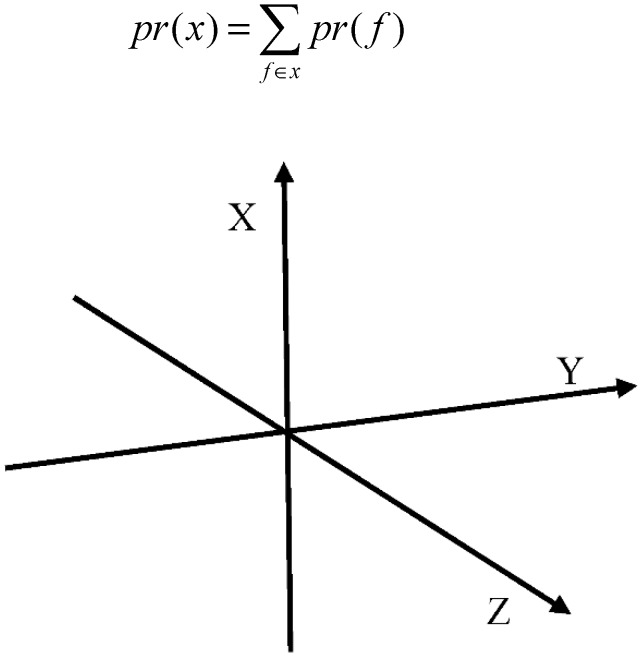
3-D subspaces model.

The quantum probability framework which relies on linear algebra of Hilbert space can be thought a generalization of classical probability. Dealing with Hilbert space lead us to use Dirac notation (bracket notation) as a sequel of quantum theory [46]. A vector of the Hilbert space is denoted by |φ〉 namely Ket, while the Bra 〈φ| is the transpose of Ket (*i.e.*, $\langle \varphi |={|\varphi \rangle }^{†}$). For simplicity, let us assume chemical-information space included three subspaces {x,y,z} as shown in Figure 1, the probability distribution over these subspaces is defined through a finite sample space $\delta =\{f_{1},\ldots ,f_{n}\}$ of cardinality *n*, where each *f_i_* corresponds to a distinct fragment, which also corresponds to chemical-information space. The latter requires orthogonal and normal basis which is known as orthonormal (*i.e.*, $\delta_{ij}$ *where* i≠j). For instance, the orthonormal basis of subspace x={|f〉 *where* f∈δ}, where this basis with norm 1. The probability distribution $pr(f_{i})$ is associated with each fragment in δ. Then, the probability of subspace *x* can be defined in space δ as:

(1) $$pr(x)=\sum_{f\in x}pr(f)$$

#### 3.1.2. Creating Reference Density

The query would be better represented by a subspace, which is spanned by the basis vector mentioned in the query. A Gleason’s theorem is an algorithm based on Hermitian operator that plays fundamental role to compute probabilities over subspaces in Hilbert space and expressed in terms of density operators. The density operator is a self-adjoint linear operator that belongs to a certain sub-class of self-adjoint operator. To each distinct fragment of the sample space is associated a one-dimensional projector $P_{f}$ corresponding to the one-dimensional subspace defined by |f〉, which is equal to |f〉〈f|. Then, all information about the probability distribution is contained into a density operator *D* defined by:

(2) $$D=\sum_{f\in \text{δ}}pr(f)p_{f}$$

where pr(f) adds up to 1.

#### 3.1.3. Complex Hilbert Space

One of a fundamental aspect of the mathematical framework of quantum physics theory is based on the presence of complex numbers. In contrast of other traditional ligand-based virtual screening models, such as vector space models and probability models rely on the use of real numbers only. Generally, the complex numbers format consist of two main parts, real part and imaginary part which can be expressed in the form x+yi, where $i=\sqrt{-1}$. While the real numbers that consist of only real part can be considered a special case. Therefore, the complex numbers that can be considered one of key concepts of the mathematical formalism of quantum provide more freedom in term of quantum mechanics theory.

The use of complex numbers in LBVS has not been used, where the representation of molecular structure only relies on real-valued vectors representation of molecular information. While the representation of information in quantum models are based on complex-valued vector representation. Although the quantum models deal with complex Hilbert space, the result of an interaction of complex vectors is always real. The first attempt that sketched out the use of complex numbers in information retrieval was proposed by [14], which stored the term frequency and the inverse term frequency in imagery and real component respectively.

This study embedded complex numbers of quantum model in LBVS based on weighting functions via three proposed ways. The global weight of any fragment which is given by a function of how many times this fragment occurs in the entire compound collection, embedded in the real component. The difference among the three techniques is the representation of imaginary part. The first representation technique use only local weight function. The local weight of any fragment is given by a function of how many times this fragment occurs in a compound/reference structure. While the second technique use both local and global weight functions. The third representation technique employed Okapi weight function which is composed of three different weight schemas, which are local, global and a new schema that produced by the integration between local and global weight schema. The Okapi weighting function with its three components previously developed by our research group as a new weighting function for a molecular similarity method based on the Bayesian network [47]. While this study modified Okapi function to embed the complex numbers of quantum model. The complex-valued representation of molecular information used by encoding fragments of molecules. While the real-valued representation also took into account as a special case of complex representation, which produced real Hilbert space. The different representations that are given by the three proposed techniques played vital role when calculate the similarity of molecules. The representation of real part can be given by Equation (3). While the imaginary component of three proposed techniques can be given by Equations (4)–(6) respectively:

(3) $$bel_{G.W.}(f_{i})=\frac{\log\left[\frac{m+0.5}{cf_{i}}\right]}{\log\left(m+1\right)}$$

(4) $$Tech_{1}(f_{i})=\frac{ff_{ij}}{ff_{ij}+0.5+1.5\times \frac{\left|c_{j}\right|}{\left|c_{avg}\right|}}$$

(5) $$Tech_{2}(f_{i})=\frac{ff_{ij}}{ff_{ij}+0.5+1.5\times \frac{\left|c_{j}\right|}{\left|c_{avg}\right|}}\times \frac{\log\left[\frac{m+0.5}{cf_{i}}\right]}{\log\left(m+1\right)}$$

(6) $$Tech_{3(Okapi)}(f_{i})=\frac{ff_{ij}}{ff_{ij}+0.5+1.5\times \frac{\left|c_{j}\right|}{\left|c_{avg}\right|}}\times \frac{\log\left[\frac{m+0.5}{cf_{i}}\right]}{\log\left(m+1\right)}\times \frac{\min(ff_{ij},ff_{ir})}{\max(ff_{ij},ff_{ir})}$$

where $ff_{ij}$ and $ff_{ir}$ are the frequency of the $i^{th}$ fragment within $j^{th}$ compound and r reference structure respectively, $cf_{i}$ is the number of compounds containing $i^{th}$ fragment, $\left|c_{j}\right|$ is the size (in terms of the number of fragments) of the $j^{th}$ compound, while $\left|c_{avg}\right|$ is the average size of all the compounds in the database, and m is the total number of compounds.

### 3.2. Tanimoto-Based Similarity Model

This model used the continuous form of the Tanimoto coefficient, which is applicable to the non-binary data of the fingerprint, *S_K,L_* is the similarity between objects or molecules *K* and *L*, which, using Tanimoto, is given by Equation (7):

(7) $$S_{SK}=\frac{\sum_{j=1}^{M}\left(w_{jk}w_{jl}\right)}{\sum_{j=1}^{M}{\left(w_{jk}\right)}^{2}+\sum_{j=1}^{M}{\left(w_{jl}\right)}^{2}-\sum_{j=1}^{M}\left(w_{jk}w_{jl}\right)}$$

For molecules described by continuous variables, the molecular space is defined by an *M × N* matrix, where entry *w*_ji_ is the value of the *j^th^* fragments (1 ≤ *j* ≤ *M*) in the *i^th^* molecule (1 ≤ *I* ≤ *N*). The origins of this coefficient can be found in [48].

### 3.3. Standard Quantum-Based Similarity Model

The probability which is computed by this model relies on quantum framework constructed of molecular compounds as shown in the previous section. The probability that a molecule is relevant to user’s reference structure is determined by projection of its vector representation onto the corresponding subspace. Let us assume the probabilistic event of molecule in Hilbert space *H* represented as a subspace $S_{m}$. A probability measure *μ* can be first defined for a *pure* chemical-information space represented as a unit vector φ, by computing the square of the length of the projection of the vector φ onto the molecular subspace $S_{m}$, (*i.e.*, ${\left\|\hat{S}\text{φ}\right\|}^{2}$). The probability can be computed by:

(8) $$\text{μ}(S_{m})=tr(d\hat{S})$$

Where tr is trace operator, and $d={\text{φ}}_{i}{\text{φ}}_{i}^{†}$ is called density operator. In general, any operator characterized by the fact that it is both positive-semi-definite (means $v^{†}pv\geq 0$ for any vector ν) and of trace 1 defines the probability distribution over the subspaces [14].

## 4. Experimental Design

The experiments were carried out using the most popular chemoinformatics databases, the MDL Drug Data Report (MDDR) [49], Maximum Unbiased Validation (MUV) [50], and Directory of Useful Decoys (DUD) [51]. All molecules in these databases were converted to Pipeline Pilot ECFC_4 (extended connectivity fingerprints and folded to size 1024 bits) [52], and these data sets have been used recently by our research group area [11,12,13,47].

The screening experiments were performed with ten reference structures selected randomly from each activity class. These structures were unified and applied on TAN and four cases of SQB similarity method. For the MDDR dataset, three data sets (MDDR-DS1, MDDR-DS2 and MDDR-DS3) with 102516 molecules were chosen. The MDDR-DS1 contains 11 activity classes, with some of the classes involving actives that are structurally homogeneous and with others involving actives that are structurally heterogeneous (*i.e.*, structurally diverse). The MDDR-DS2 data set contains 10 homogeneous activity classes, while the MDDR-DS3 data set contains 10 heterogeneous activity classes. Details of these three data sets are given in Table 1, Table 2 and Table 3. Each row of a table contains an activity class, the number of molecules belonging to the class, and the class’s diversity, which was computed as the mean pairwise Tanimoto similarity calculated across all pairs of molecules in the class using ECFC_4. The second data set, (MUV) as shown in Table 4, was reported by Rohrer and Baumann [50]. This data set contains 17 activity classes, with each class containing up to 30 actives and 15,000 inactive molecules. The diversity of the class for this dataset shows that it contains high diversity or more heterogeneous activity classes. This data set was also used in the previous study by our research group [12,47].

**Table 1:** MDDR-DS1 structure activity classes.

Activity Index	Activity Class	Active Molecules	Pairwise Similarity
31420	Renin inhibitors	1130	0.290
71523	HIV protease inhibitors	750	0.198
37110	Thrombin inhibitors	803	0.180
31432	Angiotensin II AT1 antagonists	943	0.229
42731	Substance P antagonists	1246	0.149
06233	Substance P antagonists	752	0.140
06245	5HT reuptake inhibitors	359	0.122
07701	D2 antagonists	395	0.138
06235	5HT1A agonists	827	0.133
78374	Protein kinase C inhibitors	453	0.120
78331	Cyclooxygenase inhibitors	636	0.108

**Table 2:** MDDR-DS2 structure activity classes.

Activity Index	Activity Class	Active Molecules	Pairwise Similarity
07707	Adenosine (A1) agonists	207	0.229
07708	Adenosine (A2) agonists	156	0.305
31420	Renin inhibitors 1	1300	0.290
42710	CCK agonists	111	0.361
64100	Monocyclic-lactams	1346	0.336
64200	Cephalosporins	113	0.322
64220	Carbacephems	1051	0.269
64500	Carbapenems	126	0.260
64350	Tribactams	388	0.305
75755	Vitamin D analogous	455	0.386

**Table 3:** MDDR-DS3 structure activity classes.

Activity Index	Activity Class	Active Molecules	Pairwise Similarity
09249	Muscarinic (M1) agonists	900	0.111
12455	NMDA receptor antagonists	1400	0.098
12464	Nitric oxide synthase inhibitors	505	0.102
31281	Dopamine-hydroxylase inhibitors	106	0.125
43210	Aldose reductase inhibitors	957	0.119
71522	Reverse transcriptase inhibitors	700	0.103
75721	Aromatase inhibitors	636	0.110
78331	Cyclooxygenase inhibitors	636	0.108
78348	Phospholipase A2 inhibitors	617	0.123
78351	Lipoxygenase inhibitors	2111	0.113

**Table 4:** MUV structure activity classes.

Activity Index	Activity Class	Pairwise Similarity
466	S1P1 rec. (agonists)	0.117
548	PKA (inhibitors	0.128
600	SF1 (inhibitors)	0.123
644	Rho-Kinase2 (inhibitors)	0.122
652	HIV RT-RNase (inhibitors)	0.099
689	Eph rec. A4 (inhibitors	0.113
692	SF1 (agonists)	0.114
712	HSP 90 (inhibitors) 30	0.106
713	ER-a-Coact. Bind. (inhibitors)	0.113
733	ER-b-Coact. Bind. (inhibitors)	0.114
737	ER-a-Coact. Bind. (potentiators)	0.129
810	FAK (inhibitors)	0.107
832	Cathepsin G (inhibitors)	0.151
846	FXIa (inhibitors)	0.161
852	FXIIa (inhibitors)	0.150
858	D1 rec. (allosteric modulators)	0.111
859	M1 rec. (allosteric inhibitors)	0.126

The third data set used in this study is Directory of Useful Decoys (DUD), which has been recently compiled as a benchmark data set specifically for docking methods. It was introduced by [51] and was used recently in molecular virtual screening [53] as well as molecular docking [54]. The decoys for each target were chosen specifically to fulfil a number of criteria to make them relevant and as unbiased as possible. In this study twelve subsets of DUD with 704 active compounds and 25,828 decoys were used as shown in Table 5.

This study presented SQB similarity method which deals with complex and real Hilbert space. The complex Hilbert space generated by three different proposed techniques (*i.e.*, SQB-Complex Tech. 1, SQB-Complex Tech. 2, and SQB-Complex Tech. 3), while the real Hilbert space generated as a special case of complex space (*i.e.*, SQB-Real). The comparison of the retrieval results obtained by four cases of SQB method that have been compared with Tanimoto (TAN). The TAN coefficient has been used in ligand-based virtual screening for many years and is now considered a reference standard.

**Table 5:** Number of active and inactive compounds for twelve DUD sub datasets, where *N*_a_: number of active compounds; *N*_dec_: number of decoys.

No.	Data Set	Active and Inactive	
		*N*_a_	*N*_dec_
1	Fgfr1t	120	4550
2	fxa	146	5745
3	gart	40	879
4	gbp	52	2140
5	gr	78	2947
6	hivpr	62	2038
7	hivrt	43	1519
8	hmga	35	1480
9	Hsp90	37	979
10	mr	15	636
11	na	49	1874
12	pr	27	1041
Total		704	25,828

## 5. Results and Discussion

The experimental results on MDDR-DS1, MDDR-DS2, MDDR-DS3, MUV, and DUD are presented in Table 6, Table 7, Table 8, Table 9 and Table 10 respectively, using cut offs at 1% and 5%. In these Tables, the results of SQB method which used four different cases of molecular compounds representation compared with benchmark TAN are reported. The three techniques of complex space (*i.e.*, Tech.1–Tech.3) used different schema of weighting functions to embed in complex numbers format, the real Hilbert space also generated as special case of quantum space. Each row in the tables lists the recall for top 1% and 5% of the activity class, and the best recall rate in each row are shaded. The Mean rows in the tables correspond to the mean when averaged over all activity classes (the best average is bolded), and the Shaded Cells rows correspond to the total number of shaded cells for each technique across the full set of activity classes.

The recall values of MDDR-DS1, MDDR-DS2 and MDDR-DS3 that reported in Table 6, Table 7 and Table 8 respectively showed that the proposed SQB method which deals with complex Hilbert space are obviously superior to TAN especially at cutoff 1% for DS1–DS3, and for DS1 and DS2 at cutoff 5%. The complex Hilbert space generated through the re-representation of molecular compounds in term of complex numbers format via three different ways. The Okapi technique (*i.e.*, Tech. 3) which used to embed complex Hilbert space gave superior retrieval results than TAN in DS1–DS3 for top 1%. While for 5% cutoff, the representation techniques which are Tech 1 and Tech 2 provide preferable retrieval results to TAN for DS1 and DS2 respectively. In the contrast, the results of SQB method which deals with real space are slightly inferior to TAN for both cutoffs 1% and 5% for DS1 and DS3, while it outperformed TAN for DS2 at top 5%.

For MUV Dataset, Table 9 shows the results of SQB in complex and real cases as well as TAN. It is shown that SQB method with complex representation evidently gave superior retrieval results than TAN for nine activity classes for 1% and for eight activity classes for 5%, as a consequence the average Mean is better than TAN. However, the real representation of SQB method provided superior recall values in five activity classes as well as the overall Mean outperformed TAN for top 5%, but the best Mean presented by SQB was with Tech. 3 complex representation for both cutoffs. On the other hand, for the DUD dataset that was reported in Table 10, the best retrieval results for both cutoffs were also obtained by SQB with Tech. 3 complex representation for the number of activity classes, and the overall Mean. In contrast, the performance of SQB with real space representation outperformed TAN for the cutoff 1% while it was inferior to TAN for cutoff 5%. Therefore, statistical analysis was required to provide strong judgment about the performances of the proposed methods.

Some of the activity classes, such as low-diversity activity classes, may contribute disproportionately to the overall value of mean recall. Therefore, using the mean recall value as the evaluation criterion could be impartial in some methods, but not in others. To avoid this bias, the effective performances of the different methods have been further investigated based on the total number of shaded cells for each method across the full set of activity classes. This is shown in the bottom row of Table 6, Table 7, Table 8, Table 9 and Table 10. According to the total number of shaded cells in these Tables, the Tech. 3 representation of SQB method at top 1% was the best performing search across the three data sets. In contrast, at top 5% case the TAN was equal to Tech. 3 of SQB only in high-diversity data set (*i.e.*, MDDR-DS3) while for other data sets the SQB was preferable. Moreover the SQB method was superior in terms of total number of shaded cells in MUV and DUD dataset.

The Kendall W test of concordance was used for ranking the performance of the similarity methods for the MDDR, MUV and DUD datasets. Here, the values of the recall for all activity classes (11 classes for DS1, 10 classes for DS2 and DS3, 17 classes for MUV, and 12 classes for DUD) was considered as a judge ranking (raters) of the similarity methods (ranked objects). The outputs of this test are the Kendall coefficient (*W*), Chi-Square (*X*^2^) and the significance level (*p* value). Hence, the *p* value is considered as significant if *p* < 0.05, and then it is possible to give an overall ranking to the similarity methods. For instance, the value of the Kendall coefficient for DS1 in Table 11 is 0.222 while the *p* value is significant (*p* < 0.05) and the overall rankings of the similarity methods is: SQB(C./T3) > SQB(C./T1) > SQB(C./T2) > TAN > SQB(R.). In Table 11, the results of Kendall W test of top 1% for all used datasets show that the values of associated probability (*p*) are less than 0.05. This indicates that the SQB method is significant in cut-off 1% for all cases. As a consequence, the overall ranking of techniques indicates that the SQB with Tech. 3 complex representation is superior to TAN and SQB with real representation.

On the other hand, the Kendall W test results of similarity methods in case of the top 5% for three data sets are shown in Table 12 reported. The P values for MUV and DUD datasets are 0.031, and 0.0001 respectively, which indicate that the Tech. 3 representation of SQB method still outperformed other methods. In contrast of the MDDR data set, Tech. 1 and Tech. 2 representation techniques significantly outperformed other methods for DS1 and DS2. While only for MDDR-DS3, TAN provided better ranking among other methods.

**Table 6:** Retrieval results of top 1% and 5% for MDDR-DS1 dataset.

Activity Index	SQB (Complex) Tech. 1	SQB (Complex) Tech. 2	SQB (Complex) Tech. 3	SQB (Real)	TAN	SQB (Complex) Tech. 1	SQB (Complex) Tech. 2	SQB (Complex) Tech. 3	SQB (Real)	TAN
**1%**						**5%**				
31420	72.18	72.44	73.73	70.03	69.69	87.75	87.24	87.22	84.03	83.49
71523	26.33	25.41	26.84	25.58	25.94	60.16	48.48	48.7	48.65	48.92
37110	18.33	22.09	24.73	9	9.63	39.81	45.77	45.62	19.56	21.01
31432	41.61	37.2	36.66	37.34	35.82	82	70.57	70.44	76.48	74.29
42731	19.06	20.49	21.17	17.34	17.77	28.77	24.58	24.35	28.19	29.68
06233	12.45	12.26	12.49	10.75	13.87	20.96	19.04	20.04	21.04	27.68
06245	7.18	6.37	6.03	6.03	6.51	15.39	13.99	13.72	13.63	16.54
07701	10.33	10.91	11.35	8.25	8.63	26.9	25.41	26.73	21.85	24.09
06235	10.51	10.9	10.15	9.14	9.71	22.47	23.72	22.81	19.13	20.06
78374	12.46	11.77	13.08	13.65	13.69	20.95	20.73	19.56	20.55	20.51
78331	6.08	6.54	5.92	5.78	7.17	10.31	11.48	11.37	13.1	16.2
**Mean**	21.50	21.48	**22.01**	19.35	19.85	**37.77**	35.54	35.50	33.29	34.77
**Shaded cells**	2	1	5	0	3	4	2	0	0	4

**Table 7:** Retrieval results of top 1% and 5% for MDDR-DS2 dataset.

Activity Index	SQB (Complex) Tech. 1	SQB (Complex) Tech. 2	SQB (Complex) Tech. 3	SQB (Real)	TAN	SQB (Complex) Tech. 1	SQB (Complex) Tech. 2	SQB (Complex) Tech. 3	SQB (Real)	TAN
**1%**						**5%**				
07707	72.62	71.31	72.09	58.5	61.84	75.15	74.22	74.37	70.39	70.39
07708	95.87	96.06	95.68	55.61	47.03	99.87	100	99.61	64.97	56.58
31420	72.02	71.32	78.56	62.22	65.1	95.04	95.24	94.88	87.04	88.19
42710	82.18	77.45	76.82	83	81.27	91.09	93	91.09	89.18	88.09
64100	88.9	87.92	87.8	80.73	80.31	99.23	98.94	99.03	94.59	93.75
64200	63.3	70	70.18	53.13	53.84	95.18	98.93	99.38	81.34	77.68
64220	60.9	66.79	67.58	34.61	38.64	84.06	90.9	90.62	48.11	52.19
64500	67.36	78.64	79.2	29.04	30.56	83.28	92.72	92.48	47.68	44.8
64350	82.45	80.83	81.68	81.86	80.18	96.02	93.75	90.78	87.96	91.71
75755	97.6	97.91	98.02	85.4	87.56	98.17	98.39	98.37	94.07	94.82
**Mean**	78.32	79.82	**80.76**	62.41	62.63	91.70	**93.60**	93.06	76.53	75.82
**Shaded cells**	3	1	5	1	0	3	6	1	0	0

**Table 8:** Retrieval results of top 1% and 5% for MDDR-DS3 dataset.

Activity Index	SQB (Complex) Tech. 1	SQB (Complex) Tech. 2	SQB (Complex) Tech. 3	SQB (Real)	TAN	SQB (Complex) Tech. 1	SQB (Complex) Tech. 2	SQB (Complex) Tech. 3	SQB(Real)	TAN
**1%**						**5%**				
09249	10.17	10.61	10.99	9.92	12.12	18.05	18.26	17.8	21.4	24.17
12455	5.65	6.65	7.03	5.12	6.57	7.59	10.23	11.42	8.1	10.29
12464	5.04	6.17	6.92	5.56	8.17	12.78	16.09	16.79	10.56	15.22
31281	15.14	18.19	18.67	10.29	16.95	20.86	27.43	29.05	15.14	29.62
43210	5.77	6.93	6.83	5.31	6.27	11.83	13.54	14.12	14.47	16.07
71522	4.74	6.34	6.57	3.03	3.75	10.56	13.26	13.82	9.2	12.37
75721	18.44	20.14	20.38	15.24	17.32	25.1	30.13	30.61	22.27	25.21
78331	6.16	6.03	6.16	5.48	6.31	10.16	12.11	11.97	12.03	15.01
78348	8.03	8	8.99	9.67	10.15	20	21.89	21.14	22.72	24.67
78351	10.87	11.98	12.5	10.03	9.84	11.8	12.63	13.3	11.95	11.71
**Mean**	9.0	10.10	**10.50**	7.96	9.74	14.87	17.55	18.0	14.78	**18.4**
**Shaded cells**	0	1	5	0	4	0	0	5	0	5

**Table 9:** Retrieval results of top 1% and 5% for MUV dataset.

Activity Index	SQB (Complex) Tech. 1	SQB (Complex) Tech. 2	SQB (Complex) Tech. 3	SQB (Real)	TAN	SQB (Complex) Tech. 1	SQB (Complex) Tech. 2	SQB (Complex) Tech. 3	SQB (Real)	TAN
**1%**						**5%**				
466	2.41	1.03	1.38	2.41	3.1	5.17	8.28	8.62	6.9	5.86
548	8.28	10.34	11.38	7.59	8.62	22.07	24.14	24.14	21.03	22.76
600	3.79	4.48	5.52	2.41	3.79	13.1	14.83	16.21	10.34	11.38
644	7.59	8.28	8.97	7.24	7.59	14.14	17.93	17.93	17.24	17.59
652	2.76	4.14	3.79	2.07	2.76	7.59	8.97	9.66	8.62	7.93
689	3.79	5.17	4.48	2.07	3.79	8.28	11.38	11.72	8.28	9.66
692	0.69	1.03	1.38	0.69	0.69	3.79	5.17	4.83	6.21	4.83
712	3.45	4.48	5.17	4.14	4.14	9.31	12.41	11.03	16.9	10.34
713	2.76	2.76	2.76	2.41	3.1	7.59	6.55	5.86	7.24	7.24
733	3.45	4.14	4.14	1.38	3.45	9.31	8.62	8.62	8.97	8.97
737	2.41	1.72	1.72	1.38	2.41	8.97	8.62	8.28	12.41	8.28
810	1.72	2.41	1.72	2.41	2.07	7.24	10.34	11.03	10.34	6.9
832	6.21	7.24	8.28	4.48	6.55	13.1	14.83	14.83	11.38	13.1
846	10.34	12.76	12.41	8.97	9.66	25.86	25.86	26.9	23.45	28.62
852	9.66	9.31	9.66	8.62	12.41	19.31	20	20	18.62	21.38
858	1.72	1.38	1.38	3.1	1.72	5.17	6.21	6.21	7.93	5.86
859	1.72	2.07	2.41	2.07	1.38	7.93	10	8.62	10.69	8.97
**Mean**	4.27	4.86	**5.09**	3.73	4.54	11.05	12.59	**12.61**	12.15	11.74
**Shaded cells**	2	6	9	2	3	2	3	8	5	2

**Table 10:** Retrieval results of top 1% and 5% for DUD dataset.

Activity Index	SQB (Complex) Tech. 1	SQB (Complex) Tech. 2	SQB (Complex) Tech. 3	SQB (Real)	TAN	SQB (Complex) Tech. 1	SQB (Complex) Tech. 2	SQB (Complex) Tech. 3	SQB (Real)	TAN
**1%**						**5%**				
FGFR1T	2.67	2.92	2.92	2.33	2.5	6.75	6.5	7	6.17	6.67
FXA	3.15	3.36	3.36	2.26	1.92	8.84	7.74	8.29	8.08	7.88
GART	5.25	5.75	5.75	7.5	7.75	23	22.75	23.25	22.25	22.25
GBP	15.77	16.73	15.96	13.65	13.27	28.65	30.58	30.96	21.35	20.96
GR	2.18	3.46	3.21	2.31	2.31	6.79	8.21	8.46	6.67	6.41
HIVPR	4.52	2.74	3.55	3.39	3.55	13.23	10.97	11.29	11.45	11.77
HIVRT	1.86	1.86	1.86	1.63	1.63	5.58	6.74	6.98	4.65	4.88
HMGA	6.57	5.43	5.43	5.71	6.29	10.86	11.43	13.14	10.29	10.29
HSP90	3.78	4.05	4.05	2.16	1.62	9.19	9.19	8.38	8.11	8.11
MR	5.33	5.33	5.33	5.33	5.33	8.67	9.33	10	9.33	9.33
NA	3.06	4.9	5.31	2.24	2.24	6.33	9.59	9.8	4.9	5.1
PR	2.22	2.22	2.22	1.85	1.85	5.19	6.67	5.19	4.44	4.81
**Mean**	4.69	4.89	**4.91**	4.19	4.18	11.09	11.64	**11.89**	9.8	9.87
**Shaded cells**	5	7	6	1	2	3	2	9	0	0

**Table 11:** Rankings of Similarity Methods Based on Kendall W Test Results using MDDR (DS1–DS3), MUV and DUD datasets for Top 1%.

Data Set	*W*	*X*^2^	*p*	Ranking
DS1	0.222	9.808	0.043	SQB(C./T3) > SQB(C./T1) > SQB(C./T2) > TAN > SQB(R.)
DS2	0.452	18.08	0.001	SQB(C./T3) = SQB(C./T1) > SQB(C./T2) > SQB(R.) > TAN
DS3	0.483	19.356	0.0006	SQB(C./T3) > SQB(C./T2) = TAN > SQB(C./T1) > SQB(R.)
MUV	0.272	18.518	0.0009	SQB(C./T3) > SQB(C./T2) > TAN > SQB(C./T1) > SQB(R.)
DUD	0.258	12.415	0.014	SQB(C./T3) > SQB(C./T2) > SQB(C./T1) > TAN > SQB(R.)

**Table 12:** Rankings of Similarity Methods Based on Kendall W Test Results using MDDR (DS1–DS3), MUV and DUD datasets for Top 5%.

Data Set	*W*	*X*^2^	*p*	Ranking
DS1	0.525	23.127	0.0001	SQB(C./T1) > SQB(C./T2) = TAN > SQB(C./T3) > SQB(R.)
DS2	0.738	29.535	0.000006	SQB(C./T2) > SQB(C./T1) > SQB(C./T3) > SQB(R.) = TAN
DS3	0.378	15.120	0.004	TAN > SQB(C./T3) > SQB(C./T2) > SQB(R.) > SQB(C./T1)
MUV	0.155	10.588	0.031	SQB(C./T3) = SQB(C./T2) > SQB(R.) > TAN > SQB(C./T1)
DUD	0.478	22.961	0.0001	SQB(C./T3) > SQB(C./T2) > SQB(C./T1) > TAN > SQB(R.)

The results of the MDDR search shown in Table 6, Table 7 and Table 8 show that the use of complex numbers format in Hilbert space of SQB method at cut-off 1% produced the highest mean values and number of shaded cells compared with TAN and real representation of SQB method. The best *p*-value at top 1% was 0.0006 for MDDR-DS3dataset. The technique that employed Okapi function to embed the molecules in term of the complex numbers format provided the best retrieval results compared with other embedded techniques as well as real representation and TAN. It was preferable for five activity classes and average Mean for DS1–DS3. However, the mean of retrieval results for the real SQB method was slightly inferior to TAN for DS1 and DS2. On the other hand, the TAN method was preferable for average mean to SQB complex/Tech. 3 in MDDR-DS3 dataset at 5% cut-off despite the fact both methods were equivalent for shaded cells of activity classes. While the case in MDDR-DS1 dataset is reversed, the Tech. 1 complex representation of SQB method outperformed TAN in mean recall criteria despite the shaded cells for both methods were equivalent. In contrast to homogeneous dataset, the mean of real SQB method was superior to the TAN method. While the MDDR-DS2 dataset includes highly similar activities, the MUV and DUD datasets have been carefully designed to include sets of highly dissimilar actives. Most of the similarity methods as well as our proposed methods here show a very high recall rate for the low diversity dataset and very low recall for the high diversity datasets, such as MDDR-DS3, MUV and DUD used in this study.

The results of MUV and DUD datasets are shown in Table 9 and Table 10 respectively. The results of both 1% and 5% cut-off for MUV are slightly preferable for both SQB representation methods compared to benchmark TAN method. The mean retrieval of complex Tech. 3 SQB method exceeded the TAN for both cut-offs, while according to shaded cells of activity classes, the proposed methods also was superior to TAN. In contrast, the real representation of SQB method outperformed TAN at top 5%. On the other hand, for DUD dataset the complex and real representation of SQB method at both cut-offs outperformed other methods for both criteria, whether mean recall values, and higher activity class values (*i.e.*, shaded cells). Moreover, the Kendall W test for DUD dataset show the superiority of the results of complex cases of SQB method than TAN method, where the *p*-values were 0.014 for top 1% and 0.0001 for top 5%. For MUV dataset, the results of complex Tech. 3 and Tech. 2 of SQB method outperformed other methods with significant level, where *p*-values 0.0009 and 0.031 for top 1% and 5% respectively, while the preferable results of real SQB method was only at top 5% which exceed TAN method.

From the above discussion, the proposed SQB method with four different representation cases of molecules have investigated using ten cases for three popular data sets, and both cut-offs 1% and 5%. The use of complex numbers format for molecules representation proved to be superior compared with real representation and benchmark TAN method, where the complex SQB method outperformed TAN in nine cases. The best proposed technique to embed in term of complex format is that obtained by Okapi function, which outperformed other complex techniques and real SQB as well as TAN. In contrast, the real representation of SQB method was slightly preferable in three cases for MDDR-DS2 and MUV data sets at top 5% as well as DUD dataset at top 1%.

## 6. Conclusions

This study introduced first attempt to adapt the concepts of quantum theory to present as quantum-based similarity method in ligand-based virtual screening. Moreover, the use of complex numbers to re-represent molecular compounds to correspond with the mathematical quantum space has been investigated via three different proposed techniques based on weighting function. The role of complex numbers representation of molecules played vital role in efficiency of SQB method. The SQB method deals with four different spaces depend on molecular compounds representation. The results of these proposed methods show that the similarity searching was improved and the performance of these methods outperformed the Tanimoto which is considered the conventional similarity method. The superior results for three popular chemoinformatic datasets were obtained by the embedded technique which based on Okapi function to re-represent the molecular compounds.

## References

[1] Walters W.P., Stahl M.T., Murcko M.A. (1998). Virtual screening—An overview. Drug Discov. Today.

[2] Johnson M.A., Maggiora G.M. (1990). Concepts and Applications of Molecular Similarity.

[3] Ma D.L., Chan D.S.H., Leung C.H. (2011). Molecular docking for virtual screening of natural product databases. Chem. Sci..

[4] Willett P., Barnard J.M., Downs G.M. (1998). Chemical similarity searching. J. Chem. Inf. Comput. Sci..

[5] Nikolova N., Jaworska J. (2003). Approaches to measure chemical similarity—A review. QSAR Comb. Sci..

[6] Bender A., Glen R.C. (2004). Molecular similarity: A key technique in molecular informatics. Org. Biomol. Chem..

[7] Maldonado A., Doucet J.P., Petitjean M., Fan B.T. (2006). Molecular similarity and diversity in chemoinformatics: From theory to applications. Mol. Divers..

[8] Todeschini R., Consonni V., Xiang H., Holliday J., Buscema M., Willett P. (2012). Similarity coefficients for binary chemoinformatics data: Overview and extended comparison using simulated and real data sets. J. Chem. Inf. Model..

[9] Willett P. (2006). Enhancing the effectiveness of ligand-based virtual screening using data fusion. QSAR Comb. Sci..

[10] Holliday J.D., Hu C., Willett P. (2002). Grouping of coefficients for the calculation of inter-molecular similarity and dissimilarity using 2D fragment bit-strings. Comb. Chem. High Throughput Screen..

[11] Ahmed A., Abdo A., Salim N. (2012). Ligand-based virtual screening using bayesian inference network and reweighted fragments. Sci. World J..

[12] Ahmed A., Saeed F., Salim N., Abdo A. (2014). Condorcet and borda count fusion method for ligand-based virtual screening. J. Cheminform..

[13] Abdo A., Chen B., Mueller C., Salim N., Willett P. (2010). Ligand-based virtual screening using bayesian networks. J. Chem. Inf. Model..

[14] Rijsbergen C.J.V. (2004). The Geometry of Information Retrieval.

[15] Piwowarski B., Lalmas M. (2009). A Quantum-Based Model for Interactive Information Retrieval.

[16] Melucci M., van Rijsbergen K. (2011). Quantum Mechanics and Information Retrieval. Advanced Topics in Information Retrieval.

[17] Willett P. (2000). Textual and chemical information processing: Different domains but similar algorithms. Inf. Res..

[18] Todeschini R., Consonni V. (2009). Molecular Descriptors for Chemoinformatics.

[19] Bader R.F. (1990). Atoms in Molecules: A Quantum Theory.

[20] Holder A.J., Ye L., Eick J.D., Chappelow C.C. (2006). A quantum-mechanical QSAR model to predict the refractive index of polymer matrices. QSAR Comb. Sci..

[21] Karelson M., Lobanov V.S., Katritzky A.R. (1996). Quantum-chemical descriptors in QSAR/QSPR studies. Chem. Rev..

[22] McCoy E.F., Sykes M.J. (2003). Quantum-mechanical QSAR/QSPR descriptors from momentum-space wave functions. J. Chem. Inf. Comput. Sci..

[23] Estrada E., Perdomo-López I., Torres-Labandeira J.J. (2001). Combination of 2D-, 3D-connectivity and quantum chemical descriptors in QSPR. Complexation of α- and β-cyclodextrin with benzene derivatives. J. Chem. Inf. Comput. Sci..

[24] Bhattacharjee A.K., Kyle D.E., Vennerstrom J.L., Milhous W.K. (2002). A 3D QSAR pharmacophore model and quantum chemical structure-activity analysis of chloroquine (CQ)-resistance reversal. J. Chem. Inf. Comput. Sci..

[25] Pizzoni D., Mascini M., Lanzone V., Del Carlo M., di Natale C., Compagnone D. (2014). Selection of peptide ligands for piezoelectric peptide based gas sensors arrays using a virtual screening approach. Biosens. Bioelectron..

[26] Temml V., Voss C.V., Dirsch V.M., Schuster D. (2014). Discovery of new liver X receptor agonists by pharmacophore modeling and shape-based virtual screening. J. Chem. Inf. Model..

[27] El Kerdawy A., Güssregen S., Matter H., Hennemann M., Clark T. (2013). Quantum mechanics-based properties for 3D-QSAR. J. Chem. Inf. Model..

[28] Raha K., Peters M.B., Wang B., Yu N., Wollacott A.M., Westerhoff L.M., Merz K.M. (2007). The role of quantum mechanics in structure-based drug design. Drug Discov. Today.

[29] De Vivo M. (2011). Bridging quantum mechanics and structure-based drug design. Front. Biosci..

[30] Kurauchi R., Watanabe C., Fukuzawa K., Tanaka S. (2015). Novel type of virtual ligand screening on the basis of quantum-chemical calculations for protein-ligand complexes and extended clustering techniques. Comput. Theor. Chem..

[31] Junquera J., Paz Ó., Sánchez-Portal D., Artacho E. (2001). Numerical atomic orbitals for linear-scaling calculations. Phys. Rev. B.

[32] Goedecker S. (1999). Linear scaling electronic structure methods. Rev. Mod. Phys..

[33] Zhong H.J., Ma V.P.Y., Cheng Z., Chan D.S.H., He H.Z., Leung K.H., Ma D.L., Leung C.H. (2012). Discovery of a natural product inhibitor targeting protein neddylation by structure-based virtual screening. Biochimie.

[34] Liu L., Leung K., Chan D.S., Wang Y., Ma D., Leung C. (2014). Identification of a natural product-like STAT3 dimerization inhibitor by structure-based virtual screening. Cell Death Dis..

[35] Li L., Maoshuang, Zhao K., Tian A. (2001). Semi-empirical quantum chemical study on structure-activity relationship in monocyclic-β-lactam antibiotics. J. Mol. Struct.: THEOCHEM.

[36] Zhou T., Huang D., Caflisch A. (2010). Quantum mechanical methods for drug design. Curr. Top. Med. Chem..

[37] Gironés X., Carbó-Dorca R., Ponec R. (2003). Molecular basis of LFER. Modeling of the electronic substituent effect using fragment quantum self-similarity measures. J. Chem. Inf. Comput. Sci..

[38] Carbó R., Besalú E., Amat L., Fradera X. (1995). Quantum molecular similarity measures (QMSM) as a natural way leading towards a theoretical foundation of quantitative structure-properties relationships (QSPR). J. Math. Chem..

[39] Besalú E., Gallegos A., Carbó-Dorca R. (2001). Topological quantum similarity indices and their use in QSAR: Application to several families of antimalarial compounds. Commun. Math. Comput. Chem./MATCH.

[40] Carbó-Dorca R. (2001). Inward matrix products: Extensions and applications to quantum mechanical foundations of QSAR. J. Mol. Struct.: THEOCHEM.

[41] Fradera X., Amat L., Besalü E., Carbó-Dorca R. (1997). Application of molecular quantum similarity to QSAR. Quant. Struct. Act. Relatsh..

[42] Amat L., Besalú E., Carbó-Dorca R., Ponec R. (2001). Identification of active molecular sites using quantum-self-similarity measures. J. Chem. Inf. Comput. Sci..

[43] Ponec R., Amat L., Carbó-dorca R. (1999). Molecular basis of quantitative structure-properties relationships (QSPR): A quantum similarity approach. J. Comput. Aided Mol. Des..

[44] Bultinck P., Carbó-Dorca R. (2003). Molecular quantum similarity matrix based clustering of molecules using dendrograms. J. Chem. Inf. Comput. Sci..

[45] Maggiora G., Shanmugasundaram V., Bajorath J. (2004). Molecular similarity measures. Chemoinformatics.

[46] Dirac P.A.M. (1981). The Principles of Quantum Mechanics.

[47] Abdo A., Salim N. (2010). New fragment weighting scheme for the bayesian inference network in ligand-based virtual screening. J. Chem. Inf. Model..

[48] Ellis D., Furner-Hines J., Willett P. (1993). Measuring the degree of similarity between objects in text retrieval systems. Perspect. Inf. Manag..

[49] MDL Drug Data Report (MDDR). http://www.accelrys.com/.

[50] Rohrer S.G., Baumann K. (2009). Maximum unbiased validation (MUV) data sets for virtual screening based on PubChem bioactivity data. J. Chem. Inf. Model..

[51] Huang N., Shoichet B.K., Irwin J.J. (2006). Benchmarking sets for molecular docking. J. Med. Chem..

[52] (2008). Pipeline Pilot Software.

[53] Cross S., Baroni M., Carosati E., Benedetti P., Clementi S. (2010). Flap: Grid molecular interaction fields in virtual screening. Validation using the dud data set. J. Chem. Inf. Model..

[54] Repasky M.P., Murphy R.B., Banks J.L., Greenwood J.R., Tubert-Brohman I., Bhat S., Friesner R.A. (2012). Docking performance of the glide program as evaluated on the Astex and DUD datasets: A complete set of glide SP results and selected results for a new scoring function integrating watermap and glide. J. Comput. Aided Mol. Des..

